# Native Electrophoresis-Coupled Activity Assays Reveal Catalytically-Active Protein Aggregates of *Escherichia coli* β-Glucuronidase

**DOI:** 10.1371/journal.pone.0130269

**Published:** 2015-06-29

**Authors:** Gina G. Burchett, Charles G. Folsom, Kimberly T. Lane

**Affiliations:** Department of Chemistry, Radford University, Radford, VA, United States of America; ContraFect Corporation, UNITED STATES

## Abstract

β-glucuronidase is found as a functional homotetramer in a variety of organisms, including humans and other animals, as well as a number of bacteria. This enzyme is important in these organisms, catalyzing the hydrolytic removal of a glucuronide moiety from substrate molecules. This process serves to break down sugar conjugates in animals and provide sugars for metabolism in bacteria. While β-glucuronidase is primarily found as a homotetramer, previous studies have indicated that the human form of the protein is also catalytically active as a dimer. Here we present evidence for not only an active dimer of the *E*. *coli* form of the protein, but also for several larger active complexes, including an octomer and a 16-mer. Additionally, we propose a model for the structures of these large complexes, based on computationally-derived molecular modeling studies. These structures may have application in the study of human disease, as several diseases have been associated with the aggregation of proteins.

## Introduction

β-glucuronidase is a ubiquitous enzyme, found in a number of organisms, including animals and many species of bacteria [1–3]. This enzyme (EC 3.2.1.31) catalyzes the hydrolytic cleavage of a glucuronide group from substrates to produce glucuronic acid and an organic alcohol. Substrates of the enzyme are highly varied, but include glycosaminoglycans and other sugar conjugates [4].

In humans, β-glucuronidase is found in the lysosome, and is essential to the normal restructuring and turnover of molecules in the extracellular matrix [4]. Deficiencies in the human form of the protein lead to a build-up of glycosaminoglycans, such as chondroitin sulfate, dermatan sulfate, and keratin sulfate, in the lysosomes of a variety of tissues. The accumulation of these molecules leads to the fatal lysosomal storage disease Sly Syndrome (Mucopolysaccharidosis Type VII) [5,6].

The effects of β-glucuronidase on human health is not limited to activity of the human form of the enzyme. Humans are host to a variety of bacteria, including *E*. *coli*, which produces its own β-glucuronidase [2]. β-glucuronidase has a role in bacterial metabolism, releasing glucuronides from sugar conjugates for production of ATP [7]. There has been some speculation over the years, but recent studies [8] indicate that *E*. *coli* β-glucuronidase may have a role in the development of colon cancers. The activity of this enzyme has also been associated with the side effects observed with administration of CPT-11, a cancer chemotherapy pro-drug used in the treatment of several types of cancer, including colon cancer [9]. In fact, the expression and activity of β-glucuronidase is increased with CPT-11 treatment [10,11].

The human form of β-glucuronidase (UniProt P08236) consists of four identical copies of a 629-residue peptide chain, with a total molecular weight of 300 kDa (each subunit has a mass of 75 kDa) [12]. The *E*. *coli* form of the enzyme (UniProt P05804) also exists as a homotetramer [13], with 601 residues per subunit (68 kDa) and a total molecular weight of 272 kDa. A superposition of the *E*. *coli* β-glucuronidase structure on that of the human enzyme (45% amino acid sequence identity) shows a 1.4 Å root mean square deviation over 565 equivalent Cα atoms. These structures highlight the presence of four active sites in the protein, one in each subunit. Both structures are comprised of three structural motifs in each subunit: a jelly-roll β-barrel, an immunoglobulin-like fold (a distorted β-barrel), and a TIM barrel (from N- to C-terminus).

In this paper, we present evidence for several active complexes of *E*. *coli* β-glucuronidase, including an octomer (a dimer of tetramers), as well as a 16-mer (a dimer of octomers). Additionally, we demonstrate that, like the human form of the enzyme, there is an active dimeric form of the *E*. *coli* β-glucuronidase. Finally, we present a proposed structure for these larger complexes that allow for catalytic activity by all polypeptide chains in the protein. These results could have implications in human health. There are many disease states associated with protein aggregation, including a variety of amyloidosis like Alzheimer’s disease, amyotrophic lateral sclerosis, and prion disease [14]. Protein aggregation is also a critical problem in the protein-based pharmaceutical industry [15]. Knowledge about the protein-protein interactions involved in the β-glucuronidase aggregates could be used in the development of techniques to prevent the formation of other protein aggregates implicated in human health and disease.

## Materials and Methods

### Protein Expression and Purification

The pET-28a expression plasmid containing the full-length *E*. *coli* β-glucuronidase gene with an N-terminal 6x-Histidine tag [13] (Matthew Redinbo, University of North Carolina, Chapel Hill, NC) was transformed into T7-LysS competent cells (New England Biolabs, Inc., Ipswich, MA, USA), and resulting cells were grown in LB medium with vigorous shaking (in the presence of kanamycin) at 37°C until an OD600 of approximately 0.8 was reached. Expression of the gene was induced by addition of 0.5 mM isopropyl-1-thio-D-galactopyranoside, and the cell culture was incubated for another three hours at 37°C with shaking. Cells were collected by centrifugation at 4500xg at 4°C for 10 minutes in a Sorvall fixed-angle centrifuge. Cell pellets were suspended in 50mM HEPES, pH 7.4 (Buffer A) with 0.1 mM phenylmethylsulfonyl fluoride. Resuspended cells were lysed by 30 minute incubation with 0.2 mg/mL lysozyme followed by sonication (Branson Sonifier). Lysed cells were centrifuged at 20,000xg at 4°C for 30 minutes in a Sorvall fixed-angle centrifuge. The cell lysate was added to a Ni-NTA His-Trap gravity column (Thermo-Scientific, USA) (pre-equilibrated with Buffer A). The column was washed with three bed-volumes of Buffer A with 25 mM imidazole. The protein was eluted using Buffer A with 250 mM imidazole. The purification was monitored by SDS-PAGE. Prior to SDS-PAGE, all samples were treated with sodium dodecyl sulfate, β-mercaptoethanol, and heat (100°C for five minutes).

An Amicon Ultra-15 device with a 30 kDa molecular weight cutoff (EMD Millipore, Billerica, MA, USA) was used to perform a buffer exchange into 50 mM HEPES, pH 7.4 and concentrate the purified protein to approximately 10 mg/mL, as determined by Bio-Rad Protein Assay (Bio-Rad Laboratories, Hercules, CA, USA), using bovine serum albumin as the standard. The protein was stored at 4°C.

In order to test oligomerization under alternative conditions, induction of cells in one experiment was carried out for 20 hours at 20°C. The remainder of the protein expression and purification procedure was carried out in the same manner as described above.

### Native Gel Electrophoresis and In-Gel Activity Assays

SDS-PAGE (12% acrylamide/bisacrylamide) and native (5% acrylamide/bisacrylamide) electrophoresis was performed on purified β-glucuronidase samples (50, 5, 0.5, and 0.05 μg in 50 mM HEPES, pH 7.4). To minimize heat-denaturation during native electrophoresis, gels were cooled using running cold water as 150 V of electricity was applied. NativeMark unstained protein standard (Life Technologies, Grand Island, NY, USA) was used as a molecular weight standard.

After electrophoresis, gels were removed from supports, and rinsed with distilled water. The SDS gel was then stained using Bio-Safe Coomassie Stain (Bio-Rad Laboratories). The native gel was equilibrated in 50 mM HEPES, pH 7.4 by allowing gel to incubate in buffer at room temperature for fifteen minutes. The gel was then incubated with fresh solutions of 50 mM HEPES, pH 7.4 containing 100 mM 5-bromo-4-chloro-3-indolyl β-D-glucuronide, cyclohexylammonium salt (X-GlcA; Sigma-Aldrich, Saint Louis, MO, USA) (200 mg/mL stock prepared in dimethyl sulfoxide) at room temperature for 30 minutes [15–17]. The gel was rinsed with distilled water, then stained using Bio-Safe Coomassie Stain. Gel densitometry analysis was performed using ImageJ [18] software.

Further experimentation was carried out with 0.5 μg β-glucuronidase in 50 mM HEPES, pH 7.4 containing 0 to 750 mM sodium chloride. In these experiments, the protein was incubated with sodium chloride overnight prior to native electrophoresis.

### Molecular Modeling

All molecular modeling was performed using ICM-Pro 3.8–1 from Molsoft, LLC [19]. Prior to analysis or modeling, .pdb files were converted to ICM files, optimizing hydrogens and side chains. The homotetrameric structures of *E*. *coli* (PDB ID 3LPF [13]) and human (PDB ID 3HN3 [12]) β-glucuronidase were analyzed for potential protein binding sites using the “Optimal Docking Areas” (ODA) method [20]. Two dimeric forms (software memory limitations precluded the use of the tetrameric form) of the protein were docked using the “Protein-Protein Docking” method, employing a single selected epitope in each, based on the ODA results. Resulting structures were then refined using the “Refine Conformation” method.

## Results and Discussion

### Detection of catalytically-active higher-order complexes

**Fig 1:**
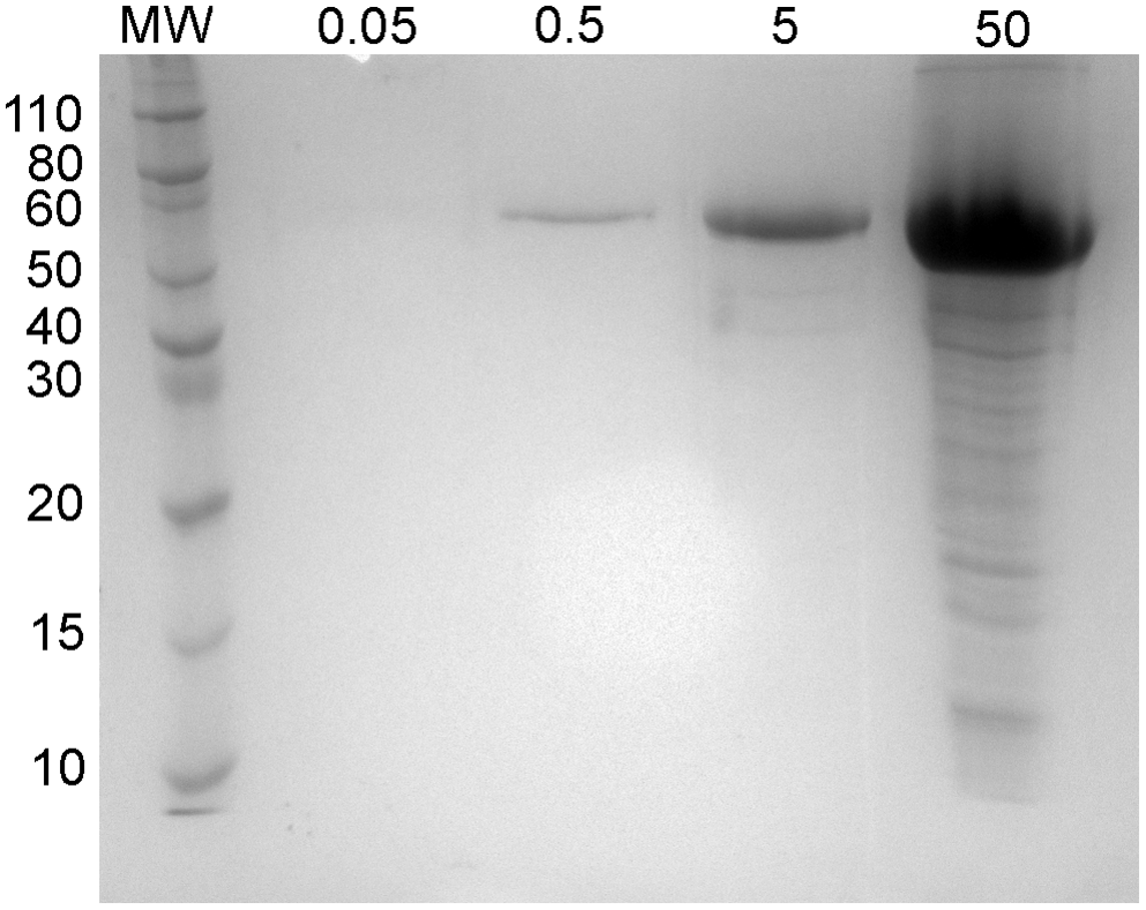
SDS-PAGE gel of *E*. *coli* β-glucuronidase enzyme. Lanes were loaded with 0.05, 0.5, 5, and 50 μg of protein.

Electrophoresis under denaturing conditions (Fig 1) confirmed the presence of a single peptide chain of approximately 68 kDa in the purified β-glucuronidase enzyme samples. In this gel, varying quantities of protein were loaded (0.05, 0.5, 5, and 50 μg, from the left). The highest quantity of protein was easily visualized (this lane was overloaded with protein), while the lowest quantity was not (no detectable protein). While optimal visualization of β-glucuronidase occurs at 0.5–5 μg of protein, lower and higher protein loads were included for comparison with native electrophoresis experiments.

**Fig 2:**
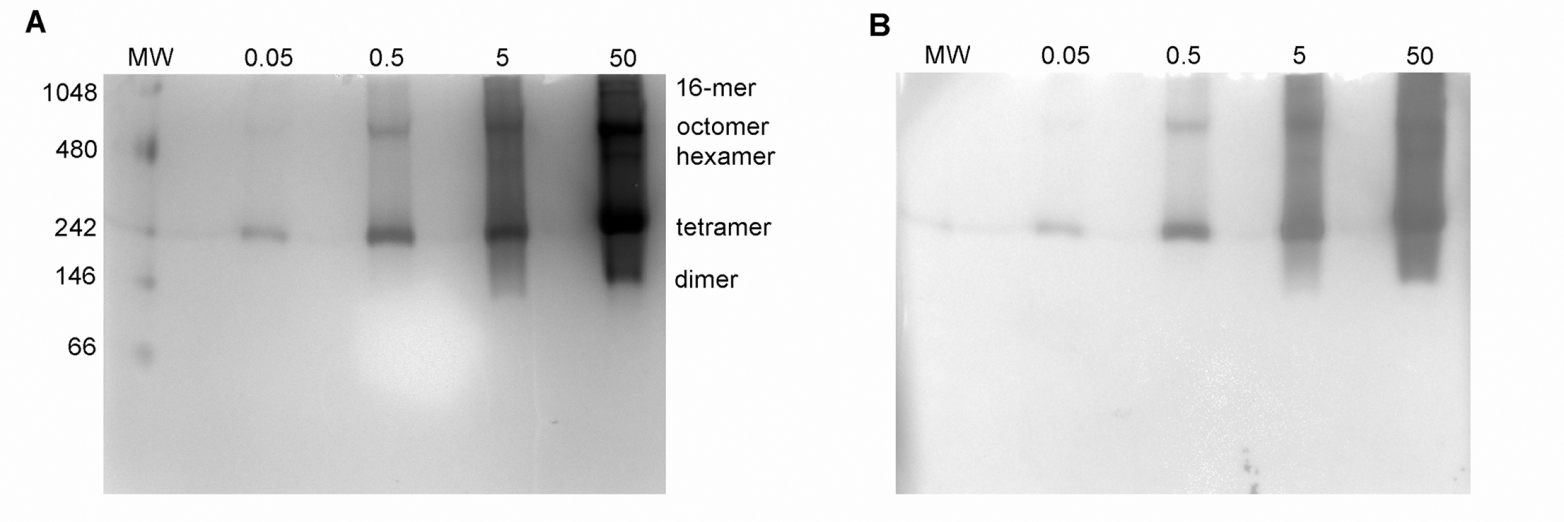
Native β-glucuronidase electrophoresis and activity assays. (A) *E*. *coli* β-glucuronidase (0.05, 0.5, 5, and 50 μg) was electrophoresed under native conditions, followed by 30-minute incubation with X-GlcA at room temperature (B). Oligomeric forms of β-glucuronidase are labeled.

Electrophoresis of purified β-glucuronidase under native conditions (Fig 2A) indicated the presence of several larger species of protein complexes, including the biologically-active tetramer (272 kDa) and octomer (544 kDa). Comparison with the results of SDS-PAGE indicates that each of the complexes observed in the native gel are oligomers of the same peptide chain. These larger octomer complex represents approximately twenty percent of the mass of each protein sample, according to densitometry analysis. This ratio (2:8, octomer:tetramer) appears to be consistent, regardless of amount of total protein in the lane. At the highest concentration of protein, additional bands that were not apparent at lower concentrations, including bands thought to represent possible dimer (136 kDa), hexamer (408 kDa) and 16-mer (1088 kDa) complexes, appear.

The results of in-gel activity assays (Fig 2B) demonstrate that these larger complexes are catalytically active, cleaving the glucuronide group from the X-GlcA substrate to produce dichlorodibromoindigo, an insoluble blue dye. This dye, during the time frame of the experiment, does not significantly diffuse away from the protein band in the gel (some product does diffuse into the surrounding solution, but does not stain the gel itself beyond the protein bands). It should be noted that the molecular weight standard used in the native electrophoresis experiments is not known to include any enzymes that would use the X-GlcA as a substrate, and will not, therefore, result in visible bands in the in-gel activity assay. These native electrophoresis experiments were repeated multiple times, each with the same result.

In addition to larger-order complexes of tetrameric β-glucuronidase, these in-gel assays indicate the presence of small amounts of an active dimer (136 kDa), as well as a hexameric complex of the dimer with a tetramer (408 kDa). Evidence for an active dimer of the human form of β-glucuronidase has been previously described [21] but this is the first indication of an active dimer of *E*. *coli* β-glucuronidase.

**Fig 3:**
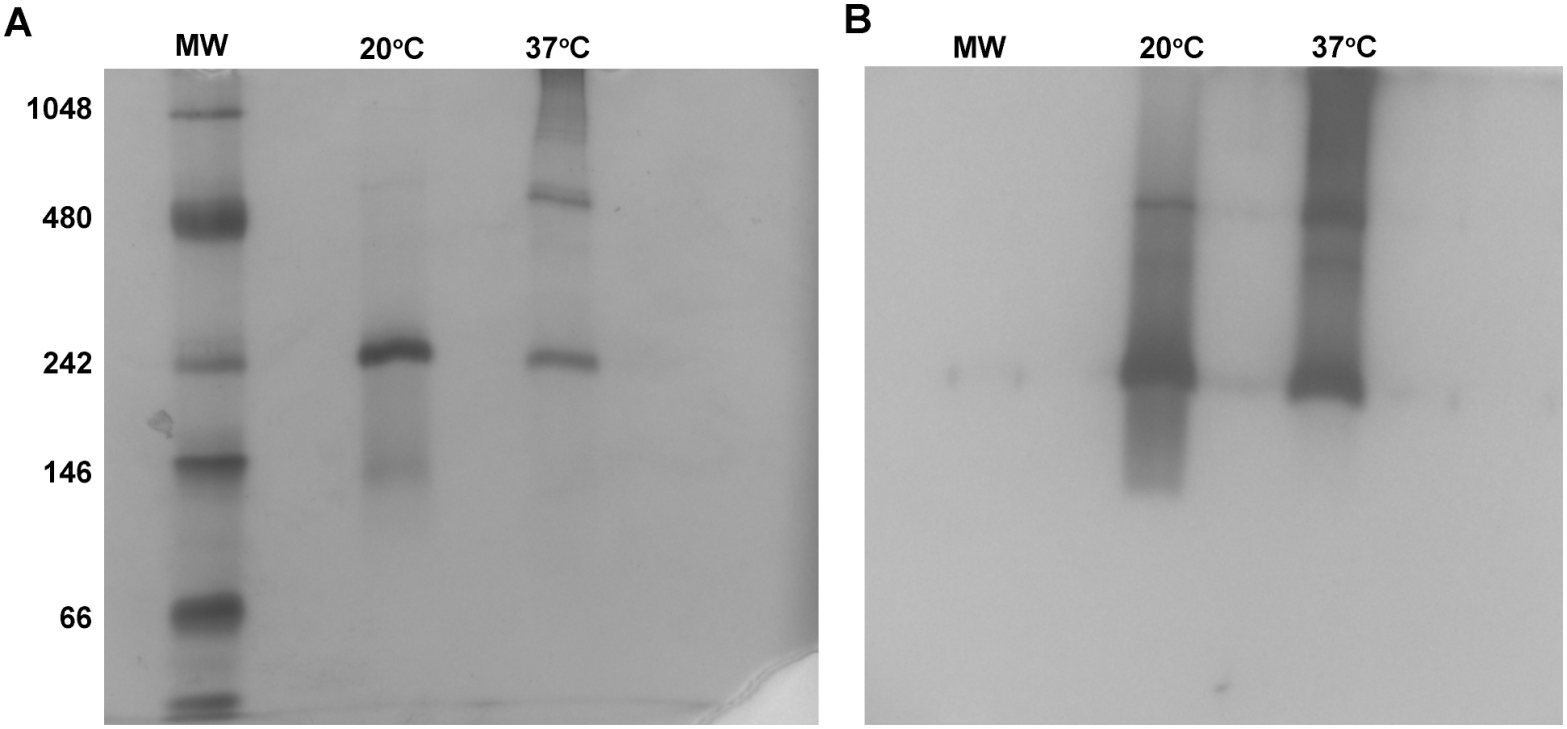
Additional native β-glucuronidase electrophoresis and activity assays. (A) *E*. *coli* β-glucuronidase induced at 20°C and 37°C was electrophoresed under native conditions, followed by 30-minute incubation with X-GlcA at room temperature (B).

To address the physiological relevance of these larger complexes, native electrophoresis was performed on β-glucuronidase under varying conditions. Addition of salt (sodium chloride, 0, 100, 250, 500, and 750 mM) to the protein sample had no effect on the observed oligomeric states of the enzyme (data not shown). Fig 3 indicates that lowering the induction temperature from 37°C to 20°C during the expression of β-glucuronidase decreases the formation of larger aggregates, including the octomer form, of the enzyme.

### Modelling the β-glucuronidase octomer

**Fig 4:**
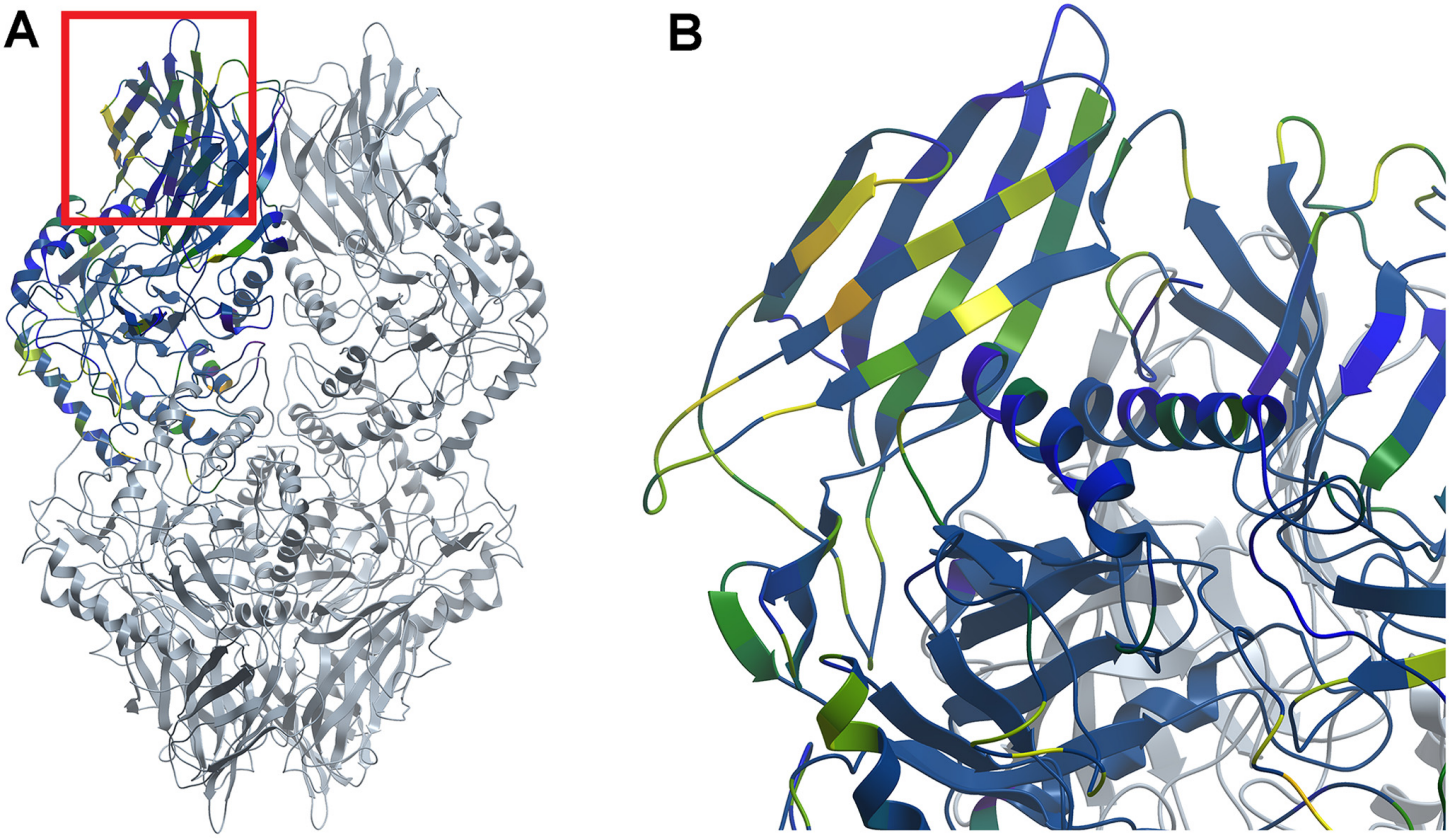
Optimal Docking Area predictions. ICM-Pro (ODA method) was used to illustrate residues of the *E*. *coli* that could be involved in protein-protein interactions. These residues are color-coded by predicted likelihood to participate in such interactions (orange = higher likelihood, yellow = some likelihood, green = lower likelihood). The predicted docking area (highlighted in red box), found in the immunoglobulin-like motif of each subunit, is shown in the context of the β-glucuronidase homotetramer (subunit displaying ODA predictions in blue, with remainder of tetramer in grey) (A). The protein structure was rotated and magnified to further illustrate the predicted docking area (B).

The molecular modeling software ICM-Pro [18] from Molsoft, LLC, uses the ODA (Optimal Docking Areas) method [20] to identify areas of potential protein interactions by comparing docking desolvation energy values calculated by atomic solvation parameters (ASP). This method identified three main areas of potential protein-protein docking on the structure of the tetrameric protein (Fig 4A). Two of these are found in the area of the structure known to be in the known subunit interfaces in the active tetramer structure, and therefore are not accessible for tetramer-tetramer interaction. The other area is defined primarily by residues to the 190–250 portion of the protein sequence (Fig 4B). The residues in the predicted protein interface, including residues V190, H192, A194, D196, C197, N198, H199, V202, W204, G232, T233, L234, V236, L241, and Q243) are located on the outer surface of an immunoglobulin-like motif. This surface was used as the epitope for protein-protein docking of two dimeric forms of the enzyme, to simplify the calculations. Each dimer was then extended to the biologically-active tetramer by superposition.

**Fig 5:**
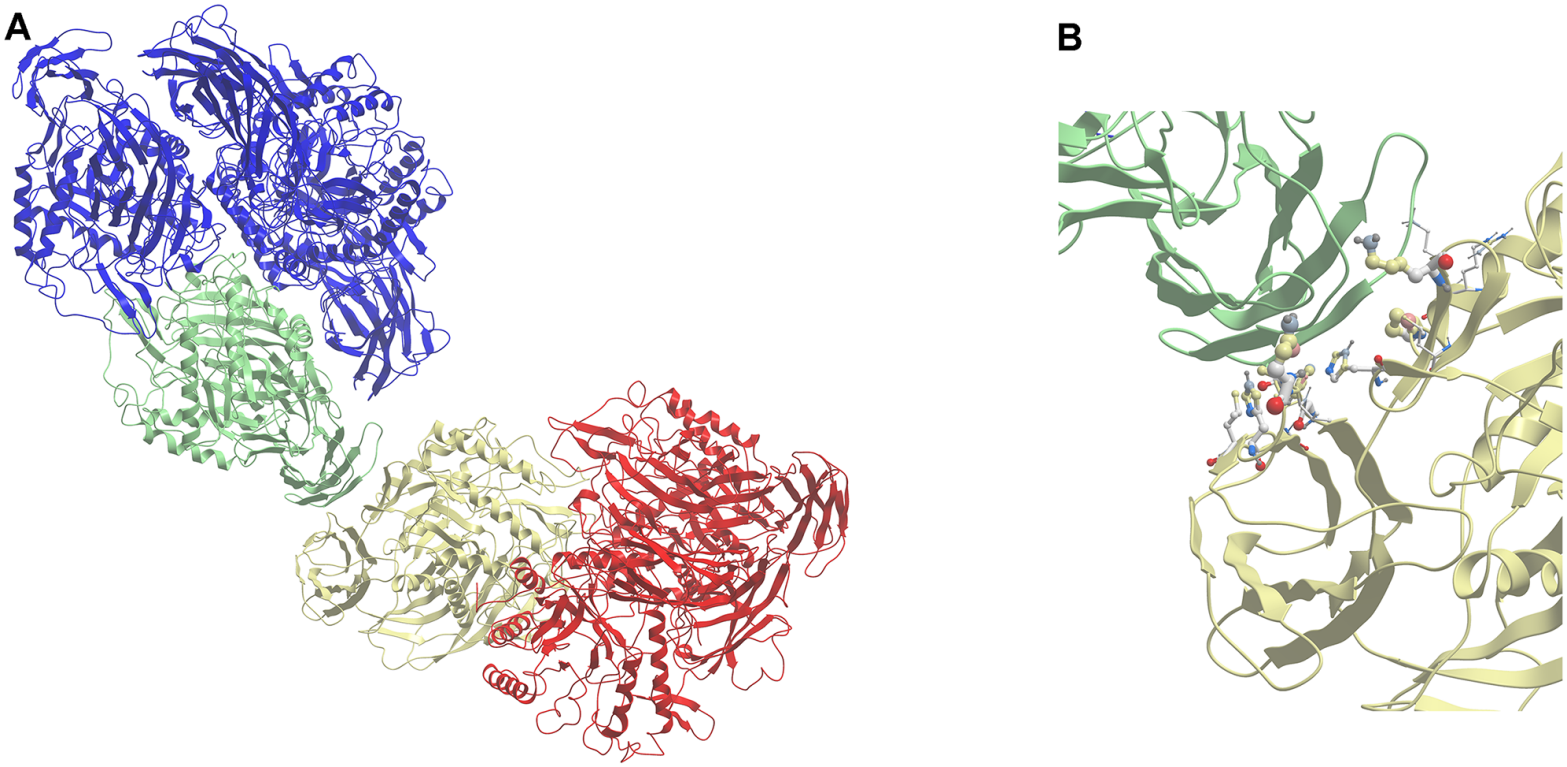
Predicted structure of β-glucuronidase octomer. (A) Overall structure, showing the entire octomer. (B) Closer look at interaction between immunoglobulin-like motifs of each subunit. Side chains involved in the interface, with degree of interaction represented by ball size (larger ball = more interaction), are included. In each illustration, tetramer A (blue) interacts with tetramer B (red) through one subunit from each (green and yellow, respectively).

The predicted structure of the octomer (Fig 5A), produced from the association of two homotetramers (therefore, known as a homodimer of homotetramers), is characterized by an association between residues in the immunoglobulin-like motif of one subunit (tetramer A) and residues in the immunoglobulin-like and TIM-barrel motifs of another (tetramer B) (Fig 5B). This interaction is similar to that observed between two jelly-roll β-barrels in one of the subunit interfaces within the homotetramer, and primarily involves nonpolar interactions. The predicted structure for the octomer allows further association with other molecules of the protein (producing 12-mer—from the binding of an octomer and tetramer or two hexamers—and 16-mer—from the binding of two octomers—complexes). Additionally, this structure leaves all active sites of the enzyme open and unblocked, with no predicted effect on catalytic activity.

It should be noted that these octomer (and larger) structures may not be observed in human β-glucuronidase. The human protein shares only a 45% sequence identity with the *E*. *coli* protein; homology within the regions proposed to be involved in the *E*. *coli* octomer formation is even lower. In fact, recent structural studies of the human enzyme indicate that Asn272 (in the proposed interface) may be modified with a glycan chain, which would prevent the structures proposed for the *E*. *coli* protein [22].

## Conclusion

These results provide the first evidence of a functional dimer in the *E*. *coli* form of the β-glucuronidase enzyme. It is unclear why β-glucuronidase has evolved to form a tetramer, when the dimeric form of the enzyme is catalytically-active. Structurally, the tetramer structure buries a large amount (approximately 4300 Å^2^, or about 20%) of surface area (primarily hydrophobic), and may serve to further stabilize the protein [12,13,21].

More importantly, these results illustrate the first evidence of catalytically-active higher-order complexes of any form of the β-glucuronidase protein. It remains to be seen if these complexes are biologically significant, although these complexes do not seem to be affected by an increase in ionic concentration to cytosolic levels. Reduction in oligomeric state at lower induction temperatures implies that aggregates of this protein may be, at least in part, a result of partial unfolding or misfolding of the protein structure [23]. The activity of these aggregates, however, demonstrates that they have a correctly-folded protein core (the active site of the enzyme). The structure of the protein may unfold slightly, exposing even more hydrophobic residues from the immunoglobulin-like folds of the protein.

These results give us further insight into the *E*. *coli* β-glucuronidase protein, and how it behaves *in vitro*. These proposed structures also give us more information about protein-protein interactions that may help to understand higher-order packing in other biological systems. There are a number of human diseases associated with protein misfolding and subsequent aggregation into long protein fibers (usually through interactions between β-structures), including amyloidosis, Alzheimer’s disease, and mad-cow disease [14]. Interestingly, the proposed interactions between tetramer A and tetramer B in the *E*. *coli* β-glucuronidase octomer involve their immunoglobulin-like motifs, which are composed of distorted β-barrels. Protein pharmaceuticals, including immunoglobulins, often aggregate, with proteins interacting through their immunoglobulin structural motifs [15]. By studying related systems, such as β-glucuronidase, we may be able to learn more about such interactions, and use the information to prevent the unwanted aggregations that can be problematic in the pharmaceutical industry.
